# Complete genome sequence of *Bacillus megaterium* strain DY294, a strain of *Bacillus* isolated from Arshan Resort in Hulunbuir

**DOI:** 10.1128/mra.00479-24

**Published:** 2024-10-21

**Authors:** Caixia Deng, Yan Zhao, Mandlaa Mandlaa, Yingsong Chen, Dezhi Yang

**Affiliations:** 1 Inner Mongolia Medical University, Hohhot, China; 2 Pharmaceutical Laboratory, International Mongolian Hospital of Inner Mongolia, Hohhot, China; 3 College of Food Science and Engineering, Inner Mongolia Agricultural University, Hohhot, China; 4 National and Local Joint Engineering Research Center for Mongolian Medicine Research and Development, Tongliao, China; SUNY College of Environmental Science and Forestry, Syracuse, New York, USA

**Keywords:** *Bacillus megaterium*, complete genome, Arshan Resort

## Abstract

The Arshan Resort is a unique mineral spring whose water is used in traditional Mongolian medicine to treat many kinds of diseases. Here, we report the genome sequence of *Bacillus megaterium* Strain DY294, a strain isolated from the Arshan water collected from Hulunbuir (117°40′ E, 48°54′ N), China.

## ANNOUNCEMENT

Three water samples were collected from the center point of the spring (117°40′6″E, 48°54′7″N) and from four points each 3 m away from the center. One hundred microliter of each sample was spread on a Rich Medium (RM) agar plate (1) and incubated at 37°C for 24 hours. Colonies were isolated by restreaking four times. All strains were identified by 16s rRNA sequencing, and strain DY294 was chosen for whole-genome sequencing (2). DY294 was cultivated in RM-broth medium for 24 hours, at 37°C, 220 rpm, and centrifuged at 8,000 rpm to collect bacteria. Genomic DNA was extracted using an SDS-based method (3). DNA library preparation for Illumina sequencing was performed using the NEBNext Ultra DNA Library Prep Kit, and 350 bp of DNA fragments were end-polished, A-tailed, and ligated with the full-length adaptor for Illumina sequencing with PCR amplification. PCR products were purified (AMPure XP system), and libraries were analyzed for size distribution by Agilent2100 Bioanalyzer and quantified using RT-PCR. Libraries for Nanopore sequencing were prepared using the SQK-LSK109 ligation sequencing kit in conjunction with the PCR-free Oxford Nanopore Technologies (ONTs) native barcode expansion kit (EXP-NBD104) with an insert size of 10 kb, and sequencing was performed without the optional shearing steps to select for long reads. The individual libraries were quantitated using a Qubit_v3.0 fluorometer (Invitrogen). Finally, the library was loaded onto an R9.4.1 flow cell and sequenced on the MinION platform (ONT). Hybrid assembly of the filtered Nanopore and Illumina reads was performed using Raven_v1.1.10, and error correction was conducted using Pilon_v1.24 (4, 5). The whole genome was sequenced using the Illumina NovaSeq PE150 and Nanopore PromethION platform.

The Illumina short reads (*n* = 184251.0 reads; 283× depth of coverage) raw data were filtered into clean reads using the SOAPnuke_v2.1.5 (6) to discard reads with an *N* ratio of >10% and low-quality bases (mass value ≤20) over 40% and corrected the genome assembly using Pilon_v1.22 (5). The resulting long-read sequences (*n* = 179845.0 reads; N_50_, 12203.0 bp; 276× depth of coverage) were assembled, with the mean read quality score of 11.4. Clean data were assembled using SPAdes_v3.10.0 (7) and integrated using CISA 1.3 (8); gapclose_v1.12 (9) to optimize the preliminary assembly result. Base-calling of the nanopore raw data was accomplished using Guppy_v6.0.6 (10), and the sequences with a Q score of <7 were discarded. Default parameters were used for all software unless otherwise specified.

Hybrid assemblies of Illumina and ONT reads were completed using Unicycler_v0.4.8 pipeline. The isolate’s assembly generated contigs of plasmids and a circular, complete chromosome. Genomes were rotated to the starting base of the *dnaA* gene. The genome achieved 100% coverage of the core genes. Genome annotation was performed by the NCBI Prokaryotic Genome Annotation Pipeline (v6.1), and the quality of assemblies and annotations was checked by BUSCO_v.5.4.6 and Quast_v.5.2.0 (11, 12). The assembled genome sequence of Strain DY294 was 6,003,706 bp in length. It consisted of one circular chromosome, 5,328,306 bp (G + C content, 37.99%), 6,426 coding gene sequences, 145 tRNA genes, and 7 plasmids.

## Data Availability

This whole-genome project has been deposited in GenBank under accession no. PRJNA1029378. The raw reads were deposited in the Sequence Read Archive under the accession numbers SRR26560369 and SRR28090477.
